# Insight into Fucoidan-Based PEGylated PLGA Nanoparticles Encapsulating Methyl Anthranilic Acid: In Vitro Evaluation and In Vivo Anti-Inflammatory Study

**DOI:** 10.3390/md20110694

**Published:** 2022-11-04

**Authors:** Dalia H. Abdelkader, Engy Elekhnawy, Walaa A. Negm, Thanaa A. El-Masry, May Almukainzi, Ahmed Zayed, Roland Ulber

**Affiliations:** 1 Pharmaceutical Technology Department, Faculty of Pharmacy, Tanta University, Tanta 31527, Egypt; 2 Pharmaceutical Microbiology Department, Faculty of Pharmacy, Tanta University, Tanta 31527, Egypt; 3 Department of Pharmacognosy, Faculty of Pharmacy, Tanta University, Tanta 31527, Egypt; 4 Department of Pharmacology and Toxicology, Faculty of Pharmacy, Tanta University, Tanta 31527, Egypt; 5 Department of Pharmaceutical Science, College Pharmacy, Princess Nourah bint Abdulrahman University, P.O. Box 84428, Riyadh 11671, Saudi Arabia; 6 Institute of Bioprocess Engineering, Technical University of Kaiserslautern, Gottlieb-Daimler-Straße 49, 67663 Kaiserslautern, Germany

**Keywords:** aqueous coating, cyclooxygenase-2, interleukins, reactive oxygen species, single emulsion solvent evaporation, tumor necrosis factor-alpha

## Abstract

A potential fucoidan-based PEGylated PLGA nanoparticles (NPs) offering a proper delivery of *N*-methyl anthranilic acid (MA, a model of hydrophobic anti-inflammatory drug) have been developed via the formation of fucoidan aqueous coating surrounding PEGylated PLGA NPs. The optimum formulation (FuP2) composed of fucoidan:m-PEG-PLGA (1:0.5 *w/w*) with particle size (365 ± 20.76 nm), zeta potential (−22.30 ± 2.56 mV), % entrapment efficiency (85.45 ± 7.41), drug loading (51.36 ± 4.75 µg/mg of NPs), % initial burst (47.91 ± 5.89), and % cumulative release (102.79 ± 6.89) has been further investigated for the anti-inflammatory in vivo study. This effect of FuP2 was assessed in rats’ carrageenan-induced acute inflammation model. The average weight of the paw edema was significantly lowered (*p* ≤ 0.05) by treatment with FuP2. Moreover, cyclooxygenase-2 and tumor necrosis factor-alpha immunostaining were decreased in FuP2 treated group compared to the other groups. The levels of prostaglandin E2, nitric oxide, and malondialdehyde were significantly reduced (*p* ≤ 0.05) in the FuP2-treated group. A significant reduction (*p* ≤ 0.05) in the expression of interleukins (IL-1*β* and IL-6) with an improvement of the histological findings of the paw tissues was observed in the FuP2-treated group. Thus, fucoidan-based PEGylated PLGA–MA NPs are a promising anti-inflammatory delivery system that can be applied for other similar drugs potentiating their pharmacological and pharmacokinetic properties.

## 1. Introduction

Inflammation is a vital immune response to various inducers like tissue injury, infections, and specific chemical agents. In this defence process, certain reactions are induced and provoked by a group of mediators controlling the inflammatory response [1]. Several pro-inflammatory mediators, like tumor necrosis factor-alpha (TNF-α), interleukin-1beta (IL-1*β*), and interleukin-6 (IL-6), are produced during the inflammatory process [2]. Such mediators initiate and augment inflammation. Additionally, various enzymes contribute to the inflammatory process, like cyclooxygenase-2 (COX-2). This is a key enzyme that can regulate the production of prostaglandins (PGs) throughout inflammation [3]. Reactive oxygen species (ROS) are additional important contributors to the inflammatory process as they have an essential role in the cellular defense system. These molecules are produced by the inflammatory cells and aggravate the oxidative stress process [4].

It has been proven that the finding of effective and safe anti-inflammatory agents is difficult. This is due to the multiple side effects of the currently used anti-inflammatory drugs like non-steroidal anti-inflammatory compounds. Furthermore, the long-term consumption of such drugs is associated with several renal, cardiac, and gastrointestinal side effects. Thus, it is crucial to find out novel and safe anti-inflammatory drugs [5]. Recently, natural products have become a crucial source for several pharmaceutical drugs, and their therapeutic potentials are being extensively explored. This is due to their multiple advantages of safety, efficacy, and biocompatibility [6].

Particularly, fucoidans are unique class of polysaccharides primarily obtained from brown algae, containing significant amounts of _L_-fucose and sulfate ester groups. Due to its multiple fascinating biological actions, fucoidans have been the subject of extensive research for the past ten years in different fields [7,8,9]. For instance, they have attracted much interest from numerous scientific disciplines, including chemistry, biology, medicine, nutrition, and pharmaceutical formulations [10,11]. The wide range of physicochemical and biological properties is thought to be the cause of all this interest [12]. Essential components of fucoidans’ molecular features include their monosaccharide composition, molecular weight, sulfation pattern, and sulfation content [13].

Fucoidans have been reported to inhibit the inflammatory processes through different pathways like selectin blockade or certain enzymes involved in the inflammation process. In addition, they revealed inhibition of the inflammatory pathologies in vivo [14]. However, several limitations have been discussed regarding fucoidans’ bioavailability and pharmacokinetic properties [15]. A minor concentration of fucoidan was absorbed via Caco-2 cells, confirmed with low uptake and distribution profile in rats, as demonstrated by Pozharitskaya et al. [16]. Hence, polymeric NPs utilizing PEGylated PLGA could be a candidate for enhancing the absorption of fucoidan via the formation of a nano delivery system potentiating the oral bioavailability of fucoidans [15,17].

Furthermore, N-methyl anthranilic acid or methyl anthranilate (MA) is commonly used in pharmaceutical preparations as a flavoring agent imparting a grape scent and flavor [18]. Asides, previous literature has discussed the anti-inflammatory effect of various anthranilic acid derivatives [19,20]. Among them, MA was chosen as a model of an anti-inflammatory drug encapsulated into NPs’ core, since it has a small molecular weight (151.16 Da) and other several drawbacks, including poor aqueous solubility concurrently with a high susceptibility to hepatic metabolism [21], making it an optimum drug of choice in this study. Hence, the aim of the present study was to investigate the possibility of incorporation of fucoidan into nanostructured composite not only for its anti-inflammatory effect but also due to its superior stability and dispersibility properties that could be offered in the developed nanosystem [15]. In addition, fucoidan-based PEGylated PLGA NPs might improve the solubility of MA and enhance its protection against liver enzymes.

## 2. Results and Discussion

### 2.1. Study the Effect of Fucoidan on m-PEG PLGA Loaded with MA

Herein, the effect of different fucoidan (purchased from Sigma-Aldrich^®^ and derived from *Fucus vesiculosus*, ≥95% pure): m-PEG PLGA ratios was investigated with regard to the physicochemical properties of NPs encapsulating MA, including particle size, zeta potential, entrapment efficiency (EE), drug loading (DL), and in vitro release behavior. The characteristics of PEGylated PLGA- loaded with MA NPs (P2) were compared with fucoidan-based PEGylated PLGA–MA NPs (FuP1, FuP2, and FuP3) in the external aqueous phase during NPs preparation, as shown in Table 1.

#### 2.1.1. Nanoparticles’ Structural Morphology

**Figure 1:**
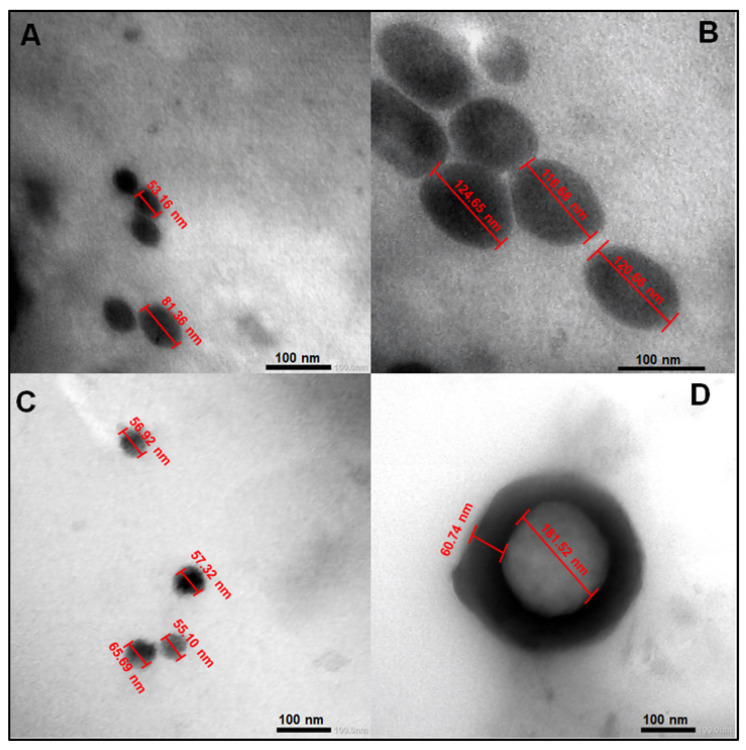
Transmission electron microscopy (TEM) images of PEGylated PLGA-MA NPs prepared with and without fucoidan. (**A**,**C**) for P2 and (**B**,**D**) for FuP2. The formula code was shown in Table 1. All data displayed were measured using the Image J program (Bethesda, MD, USA).

The effect of fucoidan on PEGylated PLGA NPs loaded with MA is clearly demonstrated in Figure 1. Fucoidan could clearly increase the average particle size of PEGylated PLGA NPs from 67.26 ± 19.94 (P2) to 120.66 ±3.98 nm (FuP2) (particle size has been calculated as a mean of numerical data displayed in Figure 1A,B), which may be explained with the formation of an additional coat covering the surface of NPs (Figure 1D). Fucoidan’s coat has a uniform spherical border with a thickness of approximately 60.74 nm surrounding the circumference of PEGylated PLGA NPs (Figure 1D). Commercial fucoidan isolated from *F. vesiculosus* (≥95%, Sigma Aldrich^®^) has an average of large molecular weight equal to 9.5 × 10^4^ Da [22], which highly contributes to producing NPs with larger particle sizes. Whereas this complete identity of the external coat could not be seen in PEGylated PLGA NPs (P2) prepared without fucoidan (Figure 1C). The particle size after utilization of fucoidan in NPs fabrication has been duplicated but with better homogeneity and uniformity that the results of dynamic light scattering (DLS) shall confirm and would be discussed in Section 2.1.3.

#### 2.1.2. Spectral Analysis via Fourier Transform Infrared (FTIR) Spectroscopy

**Figure 2:**
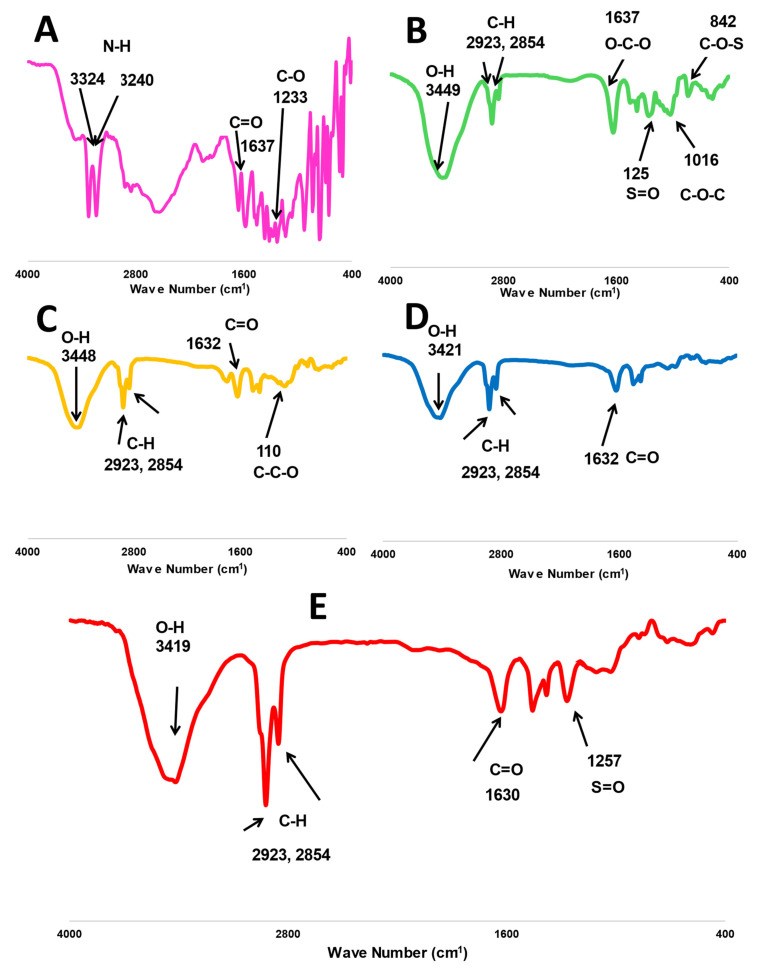
FTIR spectra of investigated formula, including (**A**) free methyl anthranilate (MA), (**B**) fucoidan, (**C**) m-PEG PLGA, (**D**) P2 (PEGylated PLGA−loaded with MA NPs), and (**E**) FuP2 (fucoidan-based PEGylated PLGA−MA NPs).

The characteristic peaks of fucoidan are presented in Figure 2, where O-H, O-C-O, S=O, and C-O-C stretching bands have been located at 3449, 1637, 1259, and 1016 cm^−1^, respectively. Additionally, a bending C-O-S peak was shown at 841 cm^−1^ [12,15]. Whereas similar peaks were found in the spectrum of m-PEG-PLGA at 3448 and 1632 cm^−1^ for O-H and C=O stretching [17] and the main peak at 1100 cm^−1^ (C-C-O stretching), indicating the chemical bonding with PEG [23,24]. MA has several characteristic peaks at 3324 and 3240 for N-H stretching. The spectral bands were at 1637 and 1233 cm^−1^ for C=O and C-O, respectively [24,25]. The two stretching C-H bands at 2923 and 2854 were common peaks presented in all FTIR spectra of MA, m-PGE PLGA, fucoidan, P2, and FuP2 (Figure 2).

Generally, all MA peaks entirely disappeared in the FTIR spectrum of P2 and FuP2, confirming a perfect encapsulation of MA into PEGylated PLGA NPs. Comparing the spectrum of FuP2 with P2, significant peaks demonstrated that both fucoidan and m-PEG PLGA have shared in forming MA NPs’ coat. Stretching the S=O peak at 1257 cm^−1^ confirms the formation of fucoidan coating on the surface of PEGylated PLGA NPs. Moreover, the C=O band is located at 1632 cm^−1^ in both P2 and m-PEG-PLGA NPs, whereas a slight shift occurred at 1630 cm^−1^ in FuP2 due to the combinatory effect of fucoidan and m-PEG PLGA polymers.

#### 2.1.3. Particle Size Polydispersity Index and Surface Charge

**Figure 3:**
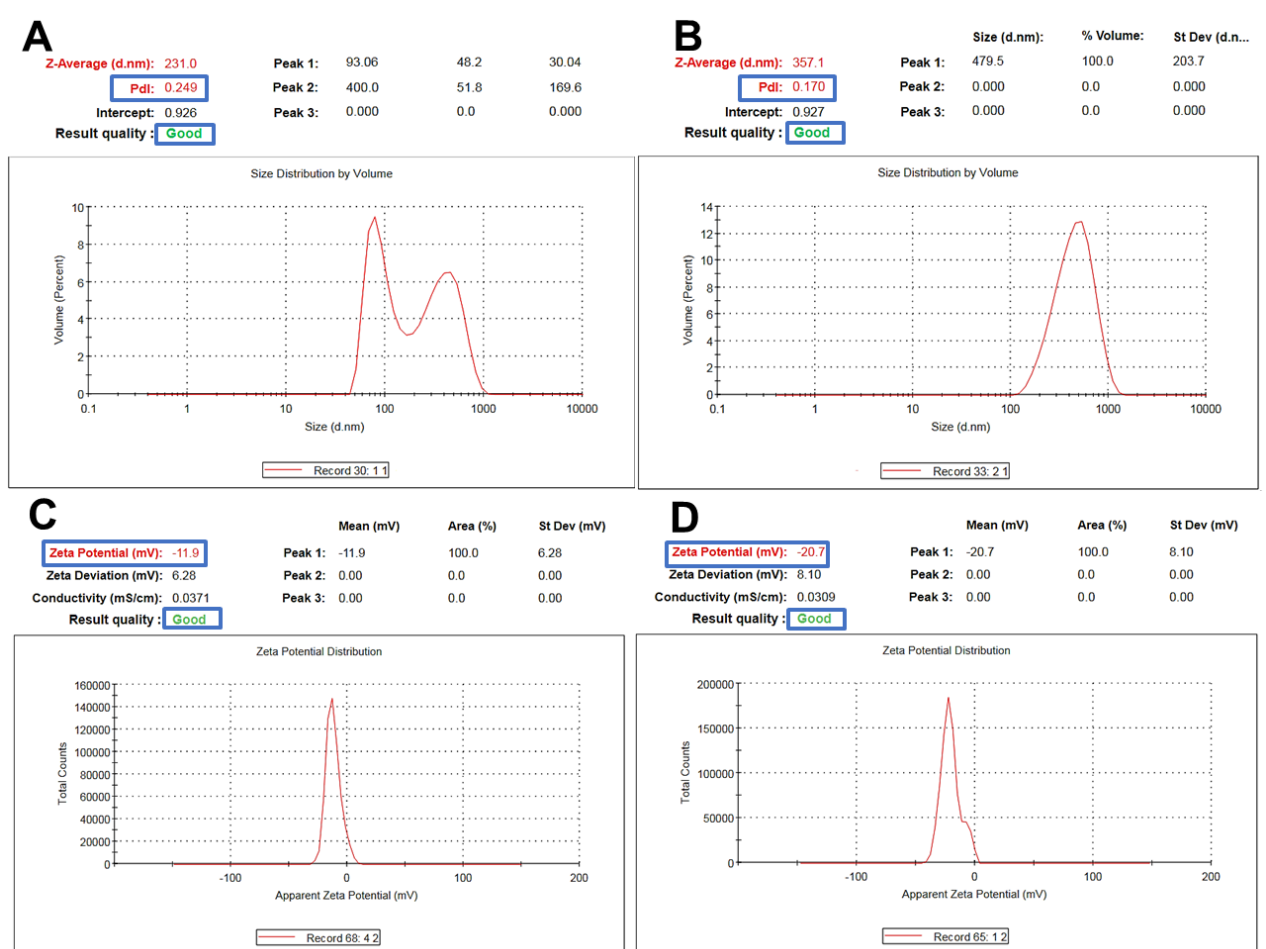
Representative charts showing particle size and zeta potential measurements of m−PEG PLGA loaded with MA. (**A**,**C**) for P2 and (**B**,**D**) for FuP2.

Fucoidan-based PEGylated PLGA NPs (FuP2) have significantly (*p* ≤ 0.05) larger particle sizes compared with PEGylated PLGA NPs prepared without fucoidan (P2), as shown in Table 1. The engagement of two polymeric matrices in NPs formulation would significantly produce a bigger particle size [26] (Figure 3A,B). Changing the fucoidan: m-PEG PLGA ratio *via* increasing m-PEG PLGA concentration significantly increased particle size (*p* ≤ 0.05). As it was displayed in Table 1, the particle size of FuP1 (270 ± 15.45 nm) has increased to 365 ± 20.76 nm in FuP2 with increasing the ratio of fucoidan: m-PEG PLGA from 1:0.25 to 1:0.50. The results obtained *via* DLS (Figure 3A,B) showed bigger particle size compared with transmission electron microscopy (TEM) (Figure 1). This might be attributed to the techniques employed while recording the measurements. TEM visualizes the real image of individual NPs dispersed in the background of the Cu grid, whereas DLS determines the hydrodynamic diameter through the scattering pattern of NPs after the direction of the light beams to follow the Brownian motion [27].

Regarding polydispersity index (PDI), fucoidan significantly influenced NPs homogeneity producing yield with uniform size distribution (Table 1, Figure 3A,B). PDI values of P2 and FuP2 were equal to 0.298 ± 0.07 and 0.172 ± 0.03, respectively. As shown in Figure 3A,B, despite fucoidan participating formulation of NPs with bigger particle sizes, it could also provide a homogenous size distribution with smaller PDI values. Fucoidan might stabilize nanoemulsion during preparation preventing the risk of aggregation, which enhances the uniform dispersibility of droplets in the emulsion background [28]. Higher m-PEG PLGA concentration substantially (*p* ≤ 0.05) increases the PDI values (Table 1). PDI values of FuP1 and FuP2 were equal to 0.115 ± 0.02 and 0.172 ± 0.03, respectively.

The key controlling factor affecting particle size and PDI is increasing m-PEG PLGA concentration [29,30]. At higher m-PEG PLGA concentration, greater viscosity of the internal organic phase will be produced, leading to the formation of more coherent oily nanodroplets, necessitating a larger shear force to be broken. Whereas we did not change the processing parameters during NPs preparation, coarser nanoemulsion with bigger particle size and inadequate homogeneity will be formulated [17,31].

Concerning zeta potential, fucoidan could substantially (*p* ≤ 0.05) increase the negative values of NPs (Table 1, Figure 3C,D). The surface charges of P2 and FuP2 were −11.50 ± 1.45 and −22.30 ± 2.56 mV, respectively, indicating that the fucoidan coating might impart more negative charge due to sulfate ester groups distributed on the fucoidan structural skeleton, potentiating the anionic feature of fucoidan [12], and therefore, enhancing higher repulsion force during NPs suspensibility leading to improved stability during preparation and storage [11]. Besides, increasing the m-PEG PLGA concentration resulted in a more negative charge surrounding NPs (Table 1) [32].

#### 2.1.4. Encapsulation Efficiency (EE) of MA into NPS’ Core

Fucoidan has a minor effect on the %EE of MA NPs (Table 1). There is no significant (*p* ≥ 0.05) difference between %EE of P2 (83.36 ± 8.45) and FuP2 (85.45 ± 7.41). The key controlling factor affecting the %EE is m-PEG PLGA concentration, a significant (*p* ≤ 0.05) greater %EE would be achieved at a higher concentration of m-PEG PLGA. The higher viscosity of the internal organic phase was attained with increasing m-PEG PLGA, leading to preventing drug molecules from escaping out to the external phase. Hence, %EE will be increased [29]. The same manner has been observed regarding the results of DL. Fucoidan has no significant effect on the amount of MA (µg) loaded in 1 mg of NPs.

#### 2.1.5. In Vitro Release Platform

**Figure 4:**
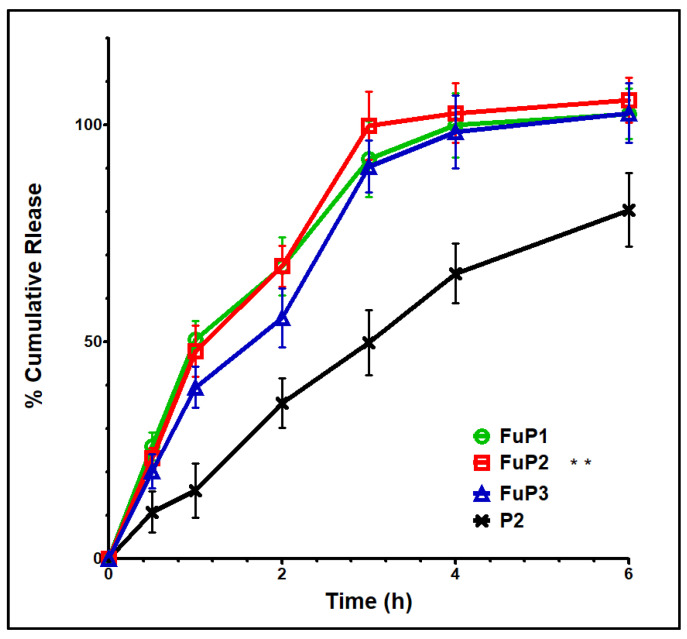
In vitro release graph of FuP1-3 and P2. Results were shown as mean ± SD. FuP2 optimum formula (double asterisks) was chosen for further investigation.

Basically, NPs with smaller particle sizes provide a better release profile with a higher initial burst and % total cumulative release over a shorter period. In our study, the incorporation of fucoidan produced NPs with bigger particle sizes. Still, this action could be over-countered by the proper aqueous solubility and well dispersibility offered by fucoidan [8]. Figure 4 shows that FuP2 displayed a release scheme with a significant (*p* ≤ 0.05) higher % initial burst (47.91 ± 5.89) at one hour and a total cumulative amount (102.79 ± 6.89) over shorter time intervals (4 h) compared with P2 (% initial burst of 35.89 ± 5.79 after 2 h, and total cumulative release of 80.46 ± 8.45 over 6 h). Denser negative charges providing proper stability with the entire external water soluble coat enhance the ease of aqueous penetration of the release media into NPs loaded with MA [33]. Regarding increasing m-PEG PLGA concentration in formulae FuP1, FuP2, and FuP3, %EE, and DL should be highly considered parallelly with the measurement of particle size during analysis of the in vitro release results [17]. It was found that FuP3 NPs have the highest values of %EE value (91.32 ± 9.23) and DL (56.37 ± 5.41), but they could not achieve the maximum % initial burst and total cumulative release due to their bigger particle size (450 ± 25.45 nm). The smaller particle size of FuP1 NPs (240 ± 12.63 nm) provides a higher % initial burst (50.56 ± 4.31) with a non-significant difference compared with FuP2 (47.91 ± 5.89). FuP2 NPs have been chosen as the optimum nano formula for in vivo anti-inflammatory implementation.

### 2.2. In Vivo Study

#### 2.2.1. Impact on the Weight of the Paw Edema

**Figure 5:**
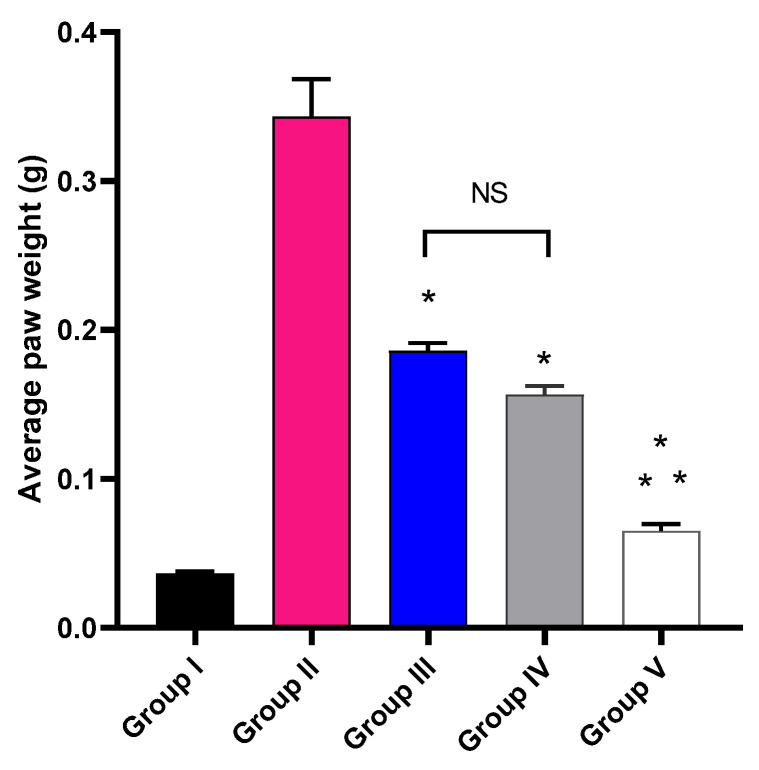
The average weight of the paw edema of the different experimental groups in comparison with group I (normal control). The symbol (*) represents a significant difference (*p* ≤ 0.05) compared with group II (model control). The symbol (**) represents a significant difference (*p* ≤ 0.05) regarding groups III (treated with MA) and IV (treated with P2). Group V (treated with FuP2) showed a significant difference (*p* ≤ 0.05). The abbreviation (NS) means a non-significant difference between groups III and IV (*p* ≥ 0.05).

The impact of MA, P2, and FuP2 treatment on the average paw edema weight was explored, as shown in Figure 5. Group V, treated with FuP2, showed a significant decrease in the average paw edema weight compared with groups II, III, and IV (*p* ≤ 0.05). In comparison with group I, group II presented a marked increase in the average paw weight with a percentage of 927.78%. Groups III and IV revealed a significant decrease in the average paw edema weight compared to group II with percentages of 48.65% and 56.76%, respectively. Remarkably, group V exhibited a substantial reduction in the average paw edema weight with a percentage of 83.78% compared to group II. It also resulted in a significant reduction regarding to groups III and IV, with percentages of 68.42% and 62.5%, respectively.

Inflammation is a process that results in the development of many pathophysiological events inducing disease progression [34]. Several animal models were established to assess the anti-inflammatory potential of different compounds. Carrageenan-induced paw edema is one of the most widely utilized animal models for evaluating the anti-inflammatory effects of many natural and synthetic agents. Carrageenan is a chemical compound able to induce the production of numerous inflammatory as well as pro-inflammatory mediators [35].

Herein, group V revealed a substantial reduction (*p* ≤ 0.05) in the paw edema weight when compared to groups III (treated with MA) and IV (treated with P2). Edema is a hallmark of the local inflammatory response resulting from fluid accumulation in the interstitial fluid in the tissues [36]. It has a detrimental impact on the function of the tissues as the accumulated fluid increases the distance of diffusion for nutrients as well as oxygen. This issue could compromise the metabolism of the cells in the swollen tissue [37].

#### 2.2.2. Histological Studies

**Figure 6:**
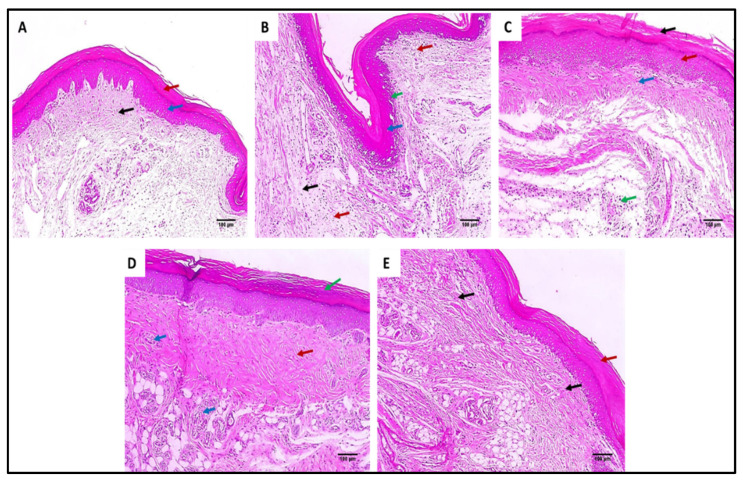
Hematoxylin and eosin (H&E)-stained paw skin sections of (**A**) Group I (normal control) shows normal skin consisting of epidermis of the average thickness (blue arrow) lined with thick keratin (red arrow) and underlying normal dermis (black arrow) (×100). (**B**) Group II (model control) showing thickened epidermis (blue arrow) with underlying granulation tissues containing sub-epidermal (green arrow) and dermal (red arrows) chronic inflammation, as well as edema (black arrow) (×100). (**C**) Group III (treated with MA) showing dermal inflammation (green arrow) surrounded by edema. The epidermis is thickened (red arrow) and covered with keratosis (black arrow) with underlying collagenosis (blue arrow) (×100). (**D**) Group IV (treated with P2) shows thickened epidermis covered with keratosis (green arrow) with underlying collagenosis (red arrow) with few inflammatory cellular infiltrates (blue arrows) (×100). (**E**) Group V (treated with FuP2) showing epidermis covered with thick keratin (red arrow) with underlying collagen fibers (black arrows) with the absence of inflammation and edema (×100).

**Figure 7:**
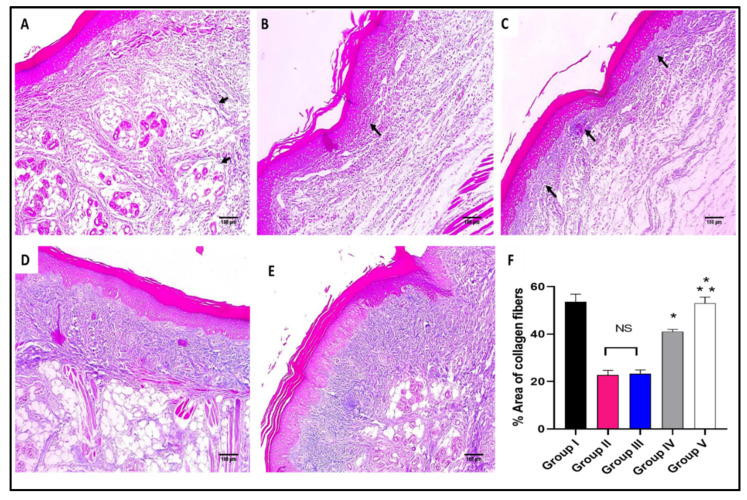
Masson’s trichrome stained paw skin sections of (**A**) Group I (normal control) shows dermal bundles of thin blue stained collagen fibers (black arrows) (×100). (**B**) Group II (model control) shows a focal increase of the collagen thickness (black arrow) (×100). (**C**) Group III (treated with MA) shows a mild collagen thickness increase (black arrow) (×100). (**D**) Group IV (treated with P2) shows a moderate increase in collagen thickness (×100). (**E**) Group V (treated with FuP2) shows a marked increase in collagen thickness (×100). (**F**) Bar chart showing the percentage of the collagen fibers in the different groups. The symbol (*) represents a significant difference (*p* ≤ 0.05) regarding group II, and (**) represents a significant difference (*p* ≤ 0.05) regarding groups III and IV. The abbreviation (NS) means a non-significant difference regarding group II (*p* ≥ 0.05).

The skin sections of the different experimental groups were stained with hematoxylin and eosin (H&E) as well as Masson’s trichrome stain, as presented in Figure 6 and Figure 7.

As revealed in Figure 7, in comparison with group I, group II presented a marked decrease in the amount of collagen fibers with a percentage of 60%. Group IV revealed a significant increase in the amount of collagen fibers compared with group II with a percentage of 86.36%. Remarkably, group V exhibited a substantial increase in the amount of collagen fibers with a percentage of 150% in comparison with group II. It also resulted in a significant increase in the amount of collagen fibers regarding to groups III and IV, with percentages of 120% and 34.15%, respectively.

#### 2.2.3. Immuno-Histochemical Studies

**Figure 8:**
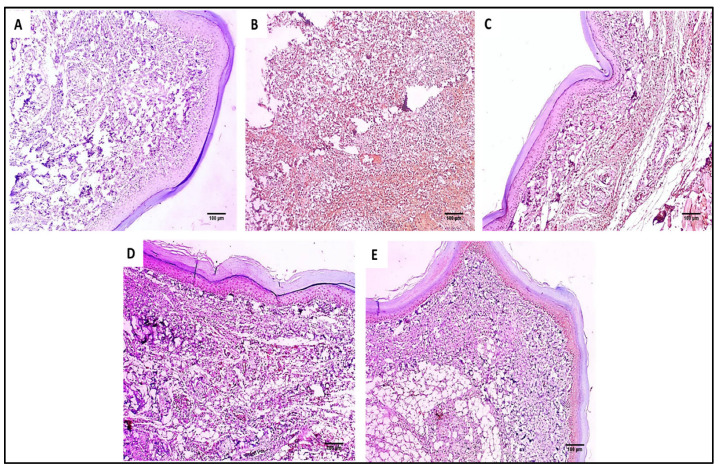
COX-2 immunostained paw skin sections of (**A**) Group I (normal control) show negative COX-2 immunostaining with a score of 0 (×100). (**B**) Group II (model control) showed strong positive COX-2 immunostaining with a score of 3 (×100). (**C**) Group III (treated with MA) showed moderate positive COX-2 immunostaining with a score of 2 (×100). (**D**) Group IV (treated with P2) showed moderate positive COX-2 immunostaining with a score of 2 (×100). (**E**) Group V (treated with FuP2) showed mild positive COX-2 immunostaining with a score of 1 (×100).

**Figure 9:**
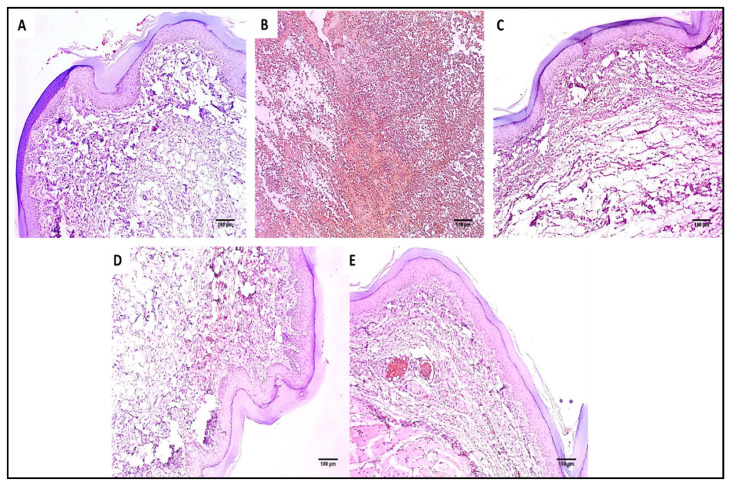
TNF-α immunostained paw skin sections of (**A**) Group I (normal control) showed negative TNF-α immunostaining with a score of 0 (×100). (**B**) Group II (model control) showed strong positive TNF-α immunostaining with a score of 3 (×100). (**C**) Group III (treated with MA) showed moderate positive TNF-α immunostaining with a score of 2 (×100). (**D**) Group IV (treated with P2) showed mild positive TNF-α immunostaining with a score of 1 (×100). (**E**) Group V (treated with FuP2) showed negative TNF-α immunostaining with a score of 0 (×100).

The COX-2 and TNF-α immunostaining of the paw skin tissues of the different experimental groups is shown in Figure 8 and Figure 9.

#### 2.2.4. Inflammatory and Oxidative Stress Markers

**Figure 10:**
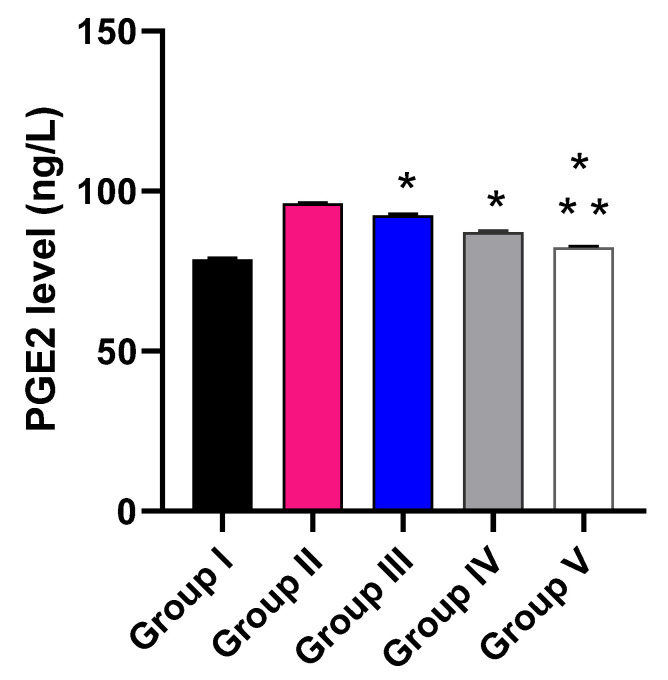
Effect of the different treatments on the level of PGE2. The symbol (*) represents a significant difference (*p* ≤ 0.05) regarding group II. The symbol (**) represents a significant difference (*p* ≤ 0.05) regarding groups III and IV. Group I is the normal control, group II is the model control, group III is the group treated with MA, group IV is the group treated with P2, and group V is the group treated with FuP2.

**Figure 11:**
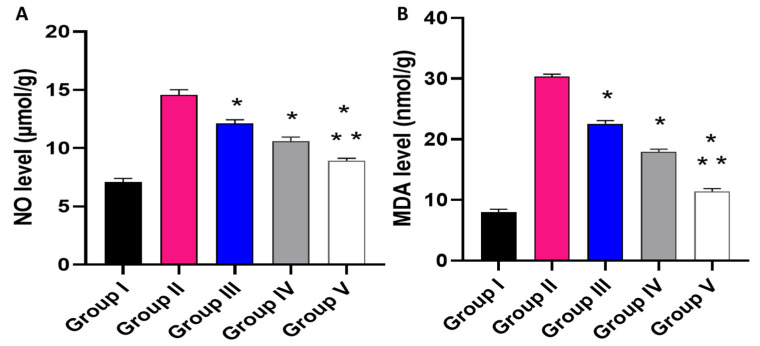
Effect of the different treatments on the levels of (**A**) NO and (**B**) MDA. The symbol (*) represents a significant difference (*p* ≤ 0.05) regarding group II. The symbol (**) represents a significant difference (*p* ≤ 0.05) regarding groups III and IV. Group I is the normal control, group II is the model control, group III is the group treated with MA, group IV is the group treated with P2, and group V is the group treated with FuP2.

The impact of the free MA, P2, and FuP2 was assessed on the inflammatory markers (PGE2) using ELISA (Figure 10) and the oxidative stress markers (NO and MDA) (Figure 11) using a colorimetric assay. Compared with group I, group II presented a marked increase in the PGE2 level with a percentage of 21.52%. Groups III and IV revealed a significant decrease in the PGE2 level compared to group II with percentages of 4.17% and 9.38%, respectively. Remarkably, group V exhibited a significant reduction in the PGE2 level with a percentage of 14.06% in comparison with group II. It also resulted in a significant decrease in the PGE2 level regarding groups III and IV, with percentages of 10.32% and 5.17%, respectively.

Regarding NO level, when compared with group I, group II presented a marked increase in the NO level with a percentage of 105.63%. Groups III and IV revealed a significant decrease in the NO level in comparison with group II with percentages of 16.44% and 26.71%, respectively. Remarkably, group V exhibited a significant reduction in the NO level with a percentage of 38.36% in comparison with group II. It also resulted in a substantial decrease in the NO level regarding groups III and IV, with 26.23% and 15.89%, respectively.

Concerning MDA level, when compared with group I, group II presented a marked increase in the MDA level with a percentage of 262.35%. Groups III and IV revealed a significant decrease in the MDA level in comparison with group II with percentages of 26.3% and 43.18%, respectively. Group V exhibited a substantial reduction in the MDA level with a percentage of 62.99% in comparison with group II. It also resulted in a substantial decrease in the MDA level regarding to groups III and IV, with percentages of 49.78% and 34.86%, respectively.

#### 2.2.5. Relative Gene Expression of IL-1*β* and IL-6

**Figure 12:**
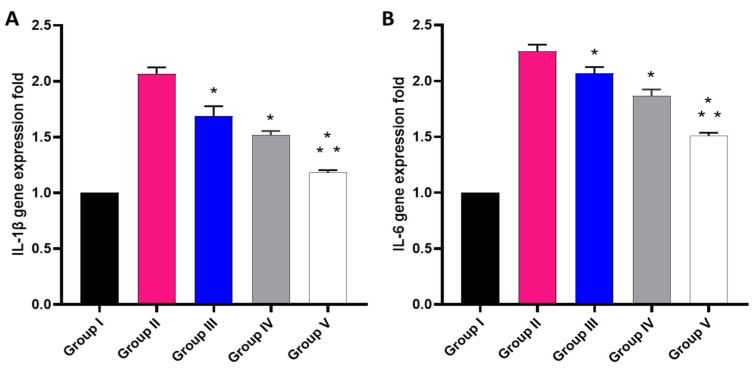
Different treatments effect on the gene expression of (**A**) IL-1*β* and (**B**) IL-6. The symbol (*) represents a significant difference (*p* ≤ 0.05) regarding group II. The symbol (**) represents a significant difference (*p* ≤ 0.05) regarding groups III and IV. Group I is the normal control, group II is the model control, group III is the group treated with MA, group IV is the group treated with P2, and group V is the group treated with FuP2.

The effect of the free MA, P2, and FuP2 was studied using qRT-PCR, as displayed in Figure 12. In comparison with group I, group II presented a marked increase in the gene expression of IL-1*β* with a percentage of 100%. Groups III and IV revealed a significant decrease in the gene expression of IL-1*β* in comparison with group II with percentages of 15% and 25%, respectively. Remarkably, group V exhibited a significant decrease in the gene expression of IL-1*β* with a percentage of 40% in comparison with group II. It also resulted in a significant reduction in the gene expression of IL-1*β* regarding groups III and IV with percentages of 24.41% and 20%, respectively. 

Concerning gene expression of IL-6, when compared with group I, group II presented a marked increase in the gene expression of IL-6 with a percentage of 120%. Groups III and IV revealed a significant decrease in the gene expression of IL-6 compared with group II with percentages of 4.55% and 13.64%, respectively. Group V exhibited a significant decline in the gene expression of IL-6 with a percentage of 31.82% in comparison with group II. It also resulted in a substantial decrease in the gene expression of IL-6 regarding to groups III and IV, with percentages of 28.57% and 21.05%, respectively. 

In the inflammation process, the macrophages and neutrophils are induced to migrate to the inflammation site. Such cells produce vast amounts of inflammatory mediators such as NO, PGE2, TNF-α, and interleukins such as IL-1*β* and IL-6. These substances stimulate a prolonged inflammatory response [38]. Many studies have explored the different biological activities of fucoidan such as its anticancer, immunomodulatory, antiviral, antioxidant, antidiabetic, anticoagulant, anti-arthritic, and anti-inflammatory impacts [38,39,40,41]. Fucoidan has the ability to lower the levels of ROS as it can lessen the production of NO by reducing the expression of nitric oxide synthase (iNOS) [42]. It is also reported that fucoidan can decrease the level of MDA, a highly reactive species formed from polyunsaturated fatty acid peroxidation [43]. Both NO and MDA are biological markers for oxidative stress [44]. The fundamental goal of the current study was to assess the efficacy of the anti-inflammatory activity of fucoidan-based PEGylated PLGA–MA NPs (FuP2) in comparison with the free MA and PEGylated PLGA-MA NPs (P2) in a carrageenan-induced edema model.

The COX-2 pathway activation and the increased PGE2 release are essential steps in the acute inflammatory response in the carrageenan model [45]. Herein, group II (carrageenan group) showed an increase in the immunostaining of COX-2 and TNF-α with a score of 3. Remarkably, this was alleviated by treatment with the fucoidan-based PEGylated PLGA–MA NPs. Moreover, the histological studies revealed that treatment with the fucoidan-based PEGylated PLGA–MA NPs resulted in the absence of inflammation and edema with a substantial increase in the collagen fibers.

The anthranilic acid derivatives are regarded as chief pharmacophores in drug discovery. Numerous anthranilic acid derivatives are utilized as therapeutic agents as they have antipyretic, analgesic, and anti-inflammatory potentials [19]. Herein, fucoidan-based PEGylated PLGA–MA NPs revealed a significant decrease (*p* ≤ 0.05) in PGE2, NO, MDA, IL-1*β*, and IL-6 compared with the free MA and PEGylated PLGA-MA NPs. This could be attributed to the potentiation of the anti-inflammatory activity by the presence of both MA and fucoidan in the FuP2 formula.

## 3. Materials and Chemicals

Fucoidan from *F. vesiculosus* ≥ 95% (Catalogue No. F8190, the average molecular weight of 9.5 × 10^4^ Da based on our previous investigation [22]), MA (Catalogue No. W268224) and poly (ethylene glycol) methyl ether-block-poly(lactide-co-glycolide) PEG average Mn 5000, PLGA average Mn 25,000, lactide: glycolide 50:50 (PEG-PLGA, Catalogue No. 799041) were purchased from Sigma-Aldrich Chemical Co. (St. Louis, MO, USA). Tween 80 was obtained from El Nasr Pharmaceutical Chemicals Co. (Cairo, Egypt). Dichloromethane (DCM) and Dimethylformamide (DMF) were supplied from Al-Gomhoria Company (Cairo, Egypt). Phosphate Buffered Saline (PBS) Tablets, Dulbecco A (OxoidTM, Thermo Fisher Scientific, Waltham, MA, USA).

## 4. Experimental Methods

MA has been employed as a model of a hydrophobic anti-inflammatory drug to investigate the validity of fucoidan–based PEGylated PLGA (FuP) NPs as a nano delivery system in terms of drug loading efficiency, in vitro release of MA from NPs’ core to the surrounding medium with sustainable manner. Additionally,, the particle size diameter, size distribution profile, and surface charge have been examined. Furthermore, compatibility between all components utilized in the fabrication of FuP NPs was analyzed using FTIR spectroscopy.

### 4.1. Preparation of Fucoidan-Based PEGylated PLGA NPs (FuP) Loaded with MA

FuP NPs loaded with MA were prepared using an adopted single emulsion/solvent evaporation method [17,29]. MA (10 mg) was dissolved in the organic phase (2 mL of DCM) comprising different concentrations of PEG PLGA (Table 1). Fucoidan (100 mg) and Tween (1% *w/w*) have been dissolved in the aqueous phase prior to the emulsification process. Under an intensive power (15 W) of sonication (Cole-Parmer Model 50 Cp T 4710 Series Ultrasonic Homogenizer, Chicago, IL, USA) with the frequency of 25 kHz, the organic phase was added to the aqueous phase (10 mL) till complete homogenization. The organic phase was evaporated under stirring for 2 h [30]. Then, the pellets were collected via washing and centrifugation (Hettich Microliter centrifuge MIKRO 220, Tuttlingen, Germany) three times and kept for further in vitro investigation.

### 4.2. In Vitro Characterization of Fucoidan-Based PEGylated PLGA NPs

#### 4.2.1. Transmission Electron Microscopy

Transmission electron microscopy (TEM, JEM-2100 E. Microscope, JEOL Ltd., Akishima, Japan) was used to visualize the formation of fucoidan coating by comparing fucoidan-based PEGylated PLGA NPs (FuP2) and P2 (The same composition prepared without fucoidan, Table 1). A few droplets of FuP2 and P2 were placed on a Cu grid coated with Carbon, kept till drying, and then analyzed under TEM. All measurements displayed on images were determined using the Image J program (Bethesda, MD, USA).

#### 4.2.2. Fourier Transform Infrared (FTIR) Spectroscopy

An appropriate amount of fucoidan, PEGylated PLGA, free MA, P2, and FuP2 were individually mixed with KBr and compressed into spherical discs for FTIR analysis. Their spectral peaks have been detected using FTIR spectrophotometer (Bruker Tensor 27, Germany) in the range of 4000–400 cm^−1^.

#### 4.2.3. Dynamic Light Scattering and Electrophoretic Mobility

The Malvern Zetasizer Nano-zs90 (Malvern Instruments Ltd., Worcestershire, UK) was used to measure the particle diameter, PDI, and zeta potential for FuP NPs (Table 1). Particle size analysis and homogeneity of size distribution were determined *via* DLS. In contrast, the surface charge was calculated through the electrophoretic mobility of NPs dispersed in the sample during analysis. All measurements were performed in triplicate, and the results were recorded as mean ± SD.

#### 4.2.4. Encapsulation Efficiency and Drug Loading

The efficiency of the fucoidan-based PEGylated PLGA nanosystem to encapsulate MA was studied indirectly via the determination of the escaped amount of MA diffusing out from nanoparticles during the centrifugation step (see Section 4.1). The supernatant was assayed using UV spectrophotometry (Evolution 300 spectrophotometer, Thermo Scientific, Waltham, MA, USA) at a maximum wavelength of 336 nm [46]. Using the following equation, %EE has been estimated [31]:

$$\%\;\mathrm{EE}=\frac{\mathrm{Initial}\;\mathrm{amount}\;\mathrm{of}\;\mathrm{drug}\;\mathrm{used}-\mathrm{amount}\;\mathrm{of}\;\mathrm{drug}\;\mathrm{remained}\;\mathrm{in}\;\mathrm{supernatant}\;}{\mathrm{Initial}\;\mathrm{amount}\;\mathrm{of}\;\mathrm{drug}\;\mathrm{used}}\times 100$$

Drug loading (DL) was determined by the digestion of nanopellets formed after the centrifugation step via the addition of DMF. The concentration of MA has been measured using UV spectrophotometry, as mentioned above, after sample’s filtration utilizing Millipore membrane filter (pore size 0.45 µm). DL was calculated using the following equation [32]:

$$\mathrm{DL}=\frac{\mathrm{Mass}\;\mathrm{of}\;\mathrm{the}\;\mathrm{drug}\;\mathrm{in}\;\mathrm{NPs}\;\left(µg\right)}{\mathrm{Mass}\;\mathrm{of}\;\mathrm{NPs}\;\left(\mathrm{mg}\right)}$$

#### 4.2.5. In Vitro Release Study

Fucoidan-based PEGylated PLGA NPs (5 mL) was packed into a dialysis bag (molecular weight cut off 12–14 kDa Fisher Scientific, Pittsburgh, PA, USA) after soaking overnight in phosphate buffer saline (PBS). Under stirring of 50 rpm and temperature equal to 37 °C, 1 mL sample was taken from the release medium (0.5% *w/v* Tween [28] in PBS, 50 mL) at different time intervals over 6 h. The samples were analyzed to determine the amount of MA released using UV spectrophotometry, as previously mentioned in Section 4.2.4.

### 4.3. In Vivo Study

#### 4.3.1. Animals

The animal house at the Faculty of Veterinary Medicine, Cairo University, Egypt, supplied us with 50 male Wistar albino rats. They weighed 180 to 210 g and were provided with a standard pellet of food in addition to filtered water at a temperature of 25 ± 2 °C and 12 h-light/dark cycle. The Research Ethical Committee (Faculty of Pharmacy, Tanta University, Egypt) accredited the in vivo protocol which was in line with the standard rules of the care and usage of laboratory animals (TP/RE/08/22P-0033).

#### 4.3.2. Carrageenan-Induced Inflammation

It was induced by a subcutaneous (SC) injection (0.2 mL) of freshly prepared carrageenan solution in normal saline in the sub-planter right hind paw. The left hind paw was not injected to preserve control [17].

#### 4.3.3. Experimental Groups

Rats were randomly classified into five groups (*n* = 10). Group I (normal control) orally received 0.9% saline. Groups II (model control) inflamed and orally received 0.9% saline. Groups III, IV, and V inflamed and orally received free MA (40 mg/kg). PEGylated PLGA-MA NPs (P2), and fucoidan-based PEGylated PLGA–MA NPs (FuP2), respectively. The amount of MA loaded in P2 (Group IV) and FuP2 (Group V) was kept being equivalent to free MA administered to Group III. After 6 h, rats were anesthetized, euthanized, and the right and left paws were cut at the same place and weighed [20]. The difference between the weights of the right and left paws was determined to calculate the average edema weight [6].

#### 4.3.4. Histological Examination

The paw tissues were preserved in formalin solution for fixation, placed in paraffin wax, sectioned (with five-micrometer thickness), and stained with H&E [47] as well as Masson’s trichrome stain [48]. Finally, these sections were inspected by a light microscope.

#### 4.3.5. Immunohistochemical Examination

The COX-2 and TNF-α expression was studied by immunostaining the paw tissues using ABclonal Technology kits (Woburn, MA, USA). The results were given scores based on the positive staining percentages. These scores are: 0 indicates the absence of positive immunostained cells, and 1 indicates the presence of 1–10% positive immunostained cells. Score 2 indicates the presence of 11–50% immunostained cells, and score 3 represents the presence of more than 50% positive immunostained cells [49].

#### 4.3.6. Enzyme-Linked Immunosorbent Assay (ELISA)

The level of prostaglandin E2 (PG-E2) in the paw tissues was detected using an ELISA kit (Creative-Biolabs, NY, USA) at 450 nm using an ELISA reader (Sunrise, Zürich, Switzerland).

#### 4.3.7. Colorimetric Determination

The levels of nitric oxide (NO) and malondialdehyde (MDA) were measured in the paw tissues using Biodiagnostic kits (Giza, Egypt) at 540 nm as described by the manufacturer.

#### 4.3.8. Quantitative Real-Time Polymerase Chain Reaction (qRT-PCR)

The inflammatory markers (IL-1*β* and IL-6) gene expression [50] was assessed in the paw tissues using qRT-PCR. The beta-actin gene [51] was the reference gene in this assay, and the used primers are revealed in Table S1.

### 4.4. Statistical Analysis

The performed experiments were conducted in triplicates and presented as the mean ± standard deviation (SD). Graph-Pad Software (prism 8, trial version) was utilized for the statistical analysis. ANOVA followed by a post-hoc test was used. The level of significance was established at *p* ≤ 0.05.

## 5. Conclusions

The developed novel formulation of fucoidan PEGylated PLGA NPs was capable of encapsulating hydrophobic MA as a drug delivery system. Fucoidan PEGylated PLGA NPs showed better homogeneity of size distribution, well dispersibility, controlled zeta potential values with good stability, and higher initial burst during the release study. Moreover, the fucoidan PEGylated PLGA–MA NPs (FuP2) potentiated the anti-inflammatory of MA in the carrageenan-induced paw edema model. This boosted anti-inflammatory impact is related to the significant decrease (*p* ≤ 0.05) in the inflammatory mediators (e.g., TNF-*α*, IL-1*β*, IL-6, and PG-E2) as well as ROS (e.g., NO and MDA). This occurred partially by the inhibition of the COX-2 pathway. Therefore, fucoidan polymeric NPs could be widely invested in further pharmaceutical studies using different polymeric materials encapsulating variable bioagents. In addition, the more effective treatment value of the developed formula compared with the free form of MA following the oral administration might indicate the better gut absorption and possible protective role against extensive liver metabolism. However, further studies shall be needed for investigating the metabolic pathway of MA in this formula.

## Figures and Tables

**Table 1:** Formula key, formulation variables, and in vitro characterization of fucoidan-based PEGylated PLGA NPs loaded with *N*-methyl anthranilic acid (MA), i.e., FuP1, FuP2, and FuP3, compared with PLGA- loaded with MA NPs (P2). Commercial fucoidan derived from *F. vesiculoisus* (≥95% pure, Sigma Aldrich^®^, St. Louis, MO, USA) was used in these formulations.

Formula Key	Fucoidan:m-PEG-PLGA (*w/w*)	Particle Size (nm)	PDI	Zeta Potential (mV)	Entrapment Efficiency (EE, %)	Drug Loading (DL, µg/mg NPs)
P2	----- *	240 ± 12.63	0.298 ± 0.07	−11.50 ± 1.45	83.36 ± 8.45	49.78 ± 3.45
FuP1	1:0.25	270 ± 15.45	0.115 ± 0.02	−16.45 ± 2.01	68.41 ± 6.85	42.89 ± 5.86
FuP2	1:0.50	365 ± 20.76	0.172 ± 0.03	−22.30 ± 2.56	85.45 ± 7.41	51.36 ± 4.75
FuP3	1:1	450 ± 25.45	0.197 ± 0.03	−25.78 ± 4.82	91.32 ± 9.23	56.37 ± 5.41

* Absence of fucoidan in P2 formula (PEGylated PLGA- loaded with MA NPs).

## Data Availability

All data is contained within the article and Supplementary Material.

## Supplementary Materials

The following supporting information can be downloaded at: https://www.mdpi.com/article/10.3390/md20110694/s1, Table S1: The sequences of the utilized primers.
