# Supplementary material for: Insight into Fucoidan-Based PEGylated PLGA Nanoparticles Encapsulating Methyl Anthranilic Acid: In Vitro Evaluation and In Vivo Anti-Inflammatory Study
Source: Mar Drugs. 2022 Nov 4;20(11):694. doi: 10.3390/md20110694 (PMC9693061; doi:10.3390/md20110694)
Supplement: Supplementary file 1 [file marinedrugs-20-00694-s001.zip › marinedrugs-1983512-SI.pdf]

Table S1. Sequences of the utilized primers

Gene	Sequence
Beta actin	Forward 5'-GTCAGGTCATCACTATCGGCAAT-3'<br>Reverse 5'- AGAGGTCTTTACGGATGTCAACGT-3'
IL-1*β*	Forward 5'-CACCTCTCAAGCAGAGCACAG-3'<br>Reverse 5'-GGGTTCCATGGTGAAGTCAAC-3'
IL-6	Forward 5'-GCCCTTCAGGAACAGCTATGA-3'<br>Reverse 5'-TGTCAACAACATCAGTCCCAAGA-3'
